# Structural problems without structural solutions? Youth leaders' perceptions of their community

**DOI:** 10.1002/ajcp.70008

**Published:** 2026-01-19

**Authors:** Linnea L. Hjelm, Aishia A. Brown, Benjamin W. Fisher, Alice Story, Nubia Bennett

**Affiliations:** ^1^ Department of Civil Society and Community Research University of Wisconsin‐Madison Louisville Kentucky USA; ^2^ Department of Health Promotion and Behavioral Sciences University of Louisville Louisville Kentucky USA; ^3^ Center for Medical Ethics and Health Policy, Baylor College of Medicine Houston Texas USA; ^4^ The Leadership Louisville Center Louisville Kentucky USA

**Keywords:** civic engagement, critical consciousness, photovoice, youth development

## Abstract

As young people explore and reflect on the conditions of their neighborhoods and communities, they can forge a critical consciousness—merging their perspectives and analysis to direct both individual and collective actions. Photovoice is a methodological tool that allows participants to document their perspectives and analysis and discuss with peers what is needed for social change. In this study, members of a local government youth program engaged in Photovoice with the ultimate goal of exploring problems and possible solutions from their points of view. Through dialogue of their selected photos, participants name a variety of structural causes of neighborhood neglect and abandonment. However, when encouraged to consider the solutions to those issues, participants predominantly identify individual or community‐level actions. We discuss the implications of this discord in the context of literature on critical consciousness and social justice youth development, with the hope of informing policy and practice decisions that can facilitate the empowerment of young people and elevate community well‐being.

## Highlights

- Youth are able to identify and name systems‐level problems that affect their community.
- When encouraged to name solutions to those issues, youth identify community/individual‐level actions.
- Youth perspectives allow insight into their progression through critical consciousness and SJYD.

## INTRODUCTION

In recent years, youth leadership programs have taken hold across the United States (Sanders et al., 2015), with the goal of both promoting healthy youth development and crafting policies informed by youth voice. At their best, these programs position youth as experts in understanding both the problems their communities face and the solutions that could address those problems, ideally providing mentorship and resources to help youth develop a critical consciousness and a social justice lens that focuses on root causes. However, young people's understanding of who is to blame for social problems and who is responsible for solving those social problems may not align (Watts & Flanagan, 2007). In this vein, the purpose of this study is to investigate how young people understand the problems facing their communities and the potential solutions to those problems. Drawing on a community‐engaged Photovoice project conducted with a youth leadership team, we demonstrate that although participants were adept at identifying structural root causes of community problems, they offered solutions that largely focused on individual‐ and community‐level actions rather than structural changes.

## MODELS OF YOUTH DEVELOPMENT

Young people need affirming settings, empowering opportunities, and supportive relationships for healthy transitions between childhood, adolescence, and adulthood (Ballard & Ozer, 2016; Bronfenbrenner & Morris, 2007). In the mid to late 20th century, youth development programs sprouted up in community spaces and local governments with the intention to intervene in the lives of young people when adults considered them likely to engage in unwanted behaviors (e.g., drug use, delinquency; Sanders et al., 2015). Whether implicitly or directly motivated by racist and classist beliefs, these interventions have traditionally targeted low‐income youth of color, essentializing them as particularly “at risk” and in need of intervention (Catalano et al., 2004; Ortega‐Williams & Harden, 2022; Travis & Leech, 2014).

The bedrock of these early programs—the *positive youth development* (PYD) framework—tends to place implicit blame on young people as their own antagonists in their pursuit of an “idealized adulthood” (Lerner et al., 2005, p. 25). This blame is exemplified in PYD's overemphasis on *individual* choices as well as its over‐valuing of behaviors that *maintain* the status quo systems that fuel disenfranchisement, two aspects heavily critiqued by youth development scholars (Ballard & Ozer, 2016, p. 224; Watts & Flanagan, 2007). In traditional PYD initiatives, little to no consideration was given to larger structural barriers and issues, like poverty and racism, that contribute to oppression and shape young people's resources, opportunities, and choices. Consequently, PYD programs are focused on transforming youth, not facilitating the empowerment of youth to transform society (Ortega‐Williams & Harden, 2022).

Alternatively, the *social justice youth development* (SJYD) model developed by Ginwright and Cammarota (2002) validates that a consideration of power and oppression is necessary to understand how a young person transitions into adulthood and engages in society. SJYD expands the PYD model by recognizing how institutions and systems enable social and health disparities that disproportionately impact those with marginalized identities (Ballard & Ozer, 2016; Ginwright & Cammarota, 2002; Watts & Flanagan, 2007). A SJYD perspective considers how young people's choices occur within and are influenced by complex sets of relationships and resources. SJYD more robustly positions youth as social actors who are shaping a more equitable future (Ginwright & James, 2002, p. 35). SJYD‐grounded programs celebrate young people's diverse identities, encouraging them to connect with peers and adult mentors with shared experiences, and allow those shared qualities to drive dialogue about community strengths and struggles (Ginwright & Cammarota, 2002, p. 34).

## YOUTH SOCIAL ANALYSIS AND CRITICAL CONSCIOUSNESS

Youth perspectives are influenced by their interpersonal relationships, education, experiences, and social/cultural norms (Bronfenbrenner & Morris, 2007; Gal, 2017; Ginwright & Cammarota, 2002). Personal experiences with marginality—along race, class, ability, and gender identities—are often the starting point for community building and organizing (Ginwright & James, 2002; Plummer et al., 2022). Alongside like‐minded peers and within SJYD‐guided youth programs, young people begin to recognize the community problems they face are derivative of oppressive systems that function because of and profit from social, political, and economic divisions (Ginwright & James, 2002). Young people question not just who/what is to *blame* for those problems but who is *responsible* for solutions, situating themselves and others in the social change landscape (Watts & Flanagan, 2007, p. 785). This burgeoning self‐awareness and social analysis are foundational to the SJYD process (Ginwright & Cammarota, 2002; Watts & Flanagan, 2007).

The tenets of SJYD are symbiotic with those of critical consciousness. Popularized by Brazilian activist and educator Paulo Freire, a critical consciousness is the ability to “read the world” and the conditions that enable oppression, and is broken down into three iterative stages: critical reflection, critical motivation, and critical action (Freire, 1970; Watts et al., 2011). People experience *critical reflection* as they learn about the systemic roots of their living conditions and make sense of their lived experiences (Diemer et al., 2016). SJYD‐oriented programming uniquely supports this introspection and analysis using dialogue, participatory research, and arts‐based activities through which young people can discern their values and connect with others (Diemer et al., 2016; Plummer et al., 2022; Watts et al., 2011). Although PYD lessons tend to focus on young people's challenges, SJYD emphasizes young people's cultural ideas, worldviews, and interests as strengths (Ginwright & James, 2002, p. 37).

Through intentional dialogue and critique of those conditions with peers, young people begin to see themselves as social actors, fostering a *critical motivation* to be part of social change. Both PYD and SJYD‐oriented initiatives might support critical motivation as they encourage youth to take accountability for their own actions and behaviors. However, although PYD might frame this responsibility as necessary for building a resilience against community harms, SJYD takes it one step further, to influence youth to consider themselves as capable of *transforming* the systems that create those harms (Ginwright & James, 2002, p. 40).

Together with like‐minded peers and supportive adults, youth can use their knowledge (critical reflection) and commitment (critical motivation) to execute *critical actions* (Diemer et al., 2021). Critical actions include both individual and collective behaviors, like protesting and communicating with public officials, that aim to dismantle oppression (Diemer et al., 2021). Unlike PYD‐based initiatives, SJYD‐motivated programs create opportunities for youth to organize, build alliances, and direct social change; these opportunities help them foster problem‐solving skills, social capital, and a consolidated social identity (Ballard & Ozer, 2016; Ginwright & James, 2002). As youth develop and social conditions evolve, they can cycle through the critical consciousness stages, as each reinforce and are reinforced by the others (Heberle et al., 2020; Watts et al., 2011).

Freire believed liberation from oppression was possible when “people develop their power to perceive critically *the way they exist* in the world *with which* and *in which* they find themselves; they come to see the world not as a static reality, but as a reality in process, in transformation” (italics in original; Freire, 1970, p. 83). Community‐organizing spaces and youth‐centered programs situated in an SJYD philosophy and encourage learning, reflection, and voice can be liberating and transformative settings for youth to build a critical consciousness. What remains under‐explored is the influence of youth development programs on how young people understand and define social problems and solutions, and how settings and activities can support this critical thinking (Kohfeldt & Langhout, 2012).

## YOUTH VOICE

In both SJYD and critical consciousness frameworks, young people exercise “control over their existence by directly engaging the conditions that shape their lives” (Ginwright & Cammarota, 2002, p. 87). As outlined in SJYD, for youth to use their social analysis to inform social action, they must have the resources and tools to transform those conditions, which include an empowered sense of voice (Cammarota, 2011). Affirming and empowering youth programs can create avenues for youth to verbalize their concerns and identify change pathways, setting a foundation for critical action (Watts et al., 2011). Indeed, participatory and youth engagement initiatives require opportunities for young people to practice critical inquiry (Kohfeldt & Langhout, 2012). SJYD and critical consciousness scholars alike highlight how individuals explore and merge their cultural, sociopolitical, and racial/ethnic identities through actions that are creative, collaborative, and executed through community coalitions (Brown et al., 2018, p. 688; Ginwright & James, 2002). Programs often report on the outcomes of youth‐engaged initiatives, though little is known about how the young people themselves perceive pathways for change and which actors and processes they believe are involved.

YPAR methods, like Photovoice, create opportunities for young people to document their lived experiences and perspectives with the goal of driving social change (Anyon et al., 2018; Ozer, 2017). For this reason, research conducted *with* historically underserved and minoritized youth and communities has the potential to highlight avenues toward social and health equity (Wallerstein & Duran, 2006). Scholars further suggest that participatory methodologies with young people are best suited to gathering their perspectives on community problems and solutions and can reveal how they traverse through the phases of critical consciousness (Watts et al., 2011) and SJYD (Ginwright & Cammarota, 2002).

## CURRENT STUDY

Considering what we now know about the difference between PYD and SJYD‐oriented youth programs and how they support critical consciousness development, this article makes two primary contributions. First, it presents a case study of a local government youth program to show how youth programming can shape how youth members perceive and define community problems and solutions. Second, we align youth testimonies with the stages of critical consciousness development and tenets of SJYD to explore how participants use their burgeoning social analysis to make sense of their world and identify what actions and alliances are needed for social change. Using Photovoice to conduct a youth‐centric social analysis, we answer the following research questions: *What are the community problems from youths' perspective? What are the solutions to community problems from youths' perspective?*

## METHOD

### Community context

The city of Louisville holds one of the largest concentrations of Black residents in Kentucky. Most of the city's residents of color are concentrated in two major areas—Buechal and the West End, which is home to about 57,000 residents—most of whom are Black (Greater Louisville Project, 2015). Black people have always shaped the development of the West End. Free Black people began buying property west of 9th street in the 1830s alongside Jewish, German, and Irish immigrants (Aubespin et al., 2011). Here, Black residents flourished in distinct neighborhoods that included arts centers, schools, newspapers, grocery stores, and other businesses through the 1950s (Aubespin et al., 2011). During desegregation, middle‐class Black families began to move away to integrated neighborhoods in other parts of the city. This left those who stayed in the West End, whether by choice or lack of opportunity, vulnerable to disinvestment, isolation, and shrinking resources as businesses closed and/or moved away (Center for Health Equity, 2017). After half a century of disinvestment, the Great Recession of 2007 saw a rise in abandoned properties and a drop in home ownership in alignment with foreclosure surges, as well as job and wage losses seen across the nation during that time.

Although the West End continues to experience the impacts of redlining, isolation, structurally racist polices, and an influx of gentrification, there are a number of grassroots and community‐based institutions committed to supporting residents in this disinvested area. Some of these efforts include Black wealth building, food justice, workforce development, reigniting a culture of public arts, and many others. As a result, and despite the structural racism, the West End continues to be a vibrant, resilient community.

### The youth implementation team

The research described here was conducted in partnership with the Louisville Metro Government Office of Safe and Healthy Neighborhoods One Love Louisville Youth Implementation Team (YIT). The YIT was made up of youth ages 14–24 with the purpose of “elevating the voices of young people on matters that impact them and their hometown” (City of Louisville Kentucky, 2019). This team worked closely with the mayor around city policies that affect youth (e.g., vaping, mental health, youth violence). YIT members were selected to represent the city's diversity along a number of domains, including race, gender, home neighborhood, and school attended. Unlike some youth leadership programs that comprise only the most academically successful students, YIT members brought a range of experiences with academic and behavioral success and struggles. In addition, members came in with varied experience with civic engagement (e.g., some youth had participated in various iterations of the YIT over the course of the program's development while others were new to the program entirely). All youth who participated in the YIT left more informed about their role in the community, with several becoming leaders of community programs and advocacy efforts following the program.

Initiatives for youth hosted by local governments offer a unique setting and access to young people who are actively exploring the role and impact of civic engagement on addressing local issues. Still, rather than focusing on transforming social structures, these programs have traditionally functioned to provide youth a structured outlet to learn new skills and become good citizens (i.e., abiding by PYD framings; Augsberger et al., 2018; Ortega‐Williams & Harden, 2022). The YIT program and its director, however, had broader intentions to support participants' social justice youth development.

To ensure a baseline of understanding, all YIT members were onboarded in a week‐long crash‐course that overviewed Louisville's civic history, government structure, and mayoral role and limitations. The training of these youth situated their lived experiences with violence, oppression, and/or privilege within a structural framework, with local and national historical roots tangled and defined by racism, classism, and redlining. Conversations during this stage of the YIT centered the question: “Do you want to do something about the community you live in?”. Youth were also introduced to youth participatory action research (YPAR) as a foundational tool for structural change and had opportunities to demonstrate what they were learning and doing through quarterly engagement with the current mayor, speaking opportunities with the metro council, and letters to the editor of the local newspaper.

### Study design

A community‐based Photovoice project was conducted in Louisville, KY during the 2019–2020 academic year with the YIT. Photovoice is a participatory research method that holds three main goals: (1) to enable people to record and reflect their community's strengths and concerns, (2) to promote critical dialogue and knowledge about important issues through large and small group discussion of photographs, and (3) to reach policymakers (Wang & Burris, 1997). We followed Delgado's (2015) nine‐stage framework for conducting Photovoice projects with youth living in urban areas: (1) pre‐project considerations (including the creation of a project advisory committee and preliminary fieldwork); (2) selection of project participants and leadership; (3) training and ongoing support (including necessary management and care of equipment and approaches toward photography); (4) assessment (needs and assets) of goals; (5) planning, scheduling, and implementation; (6) selection of images and narratives; (7) cultural final portrait/exhibition; (8) project evaluation; (9) post‐Photovoice social change action selection. However, when the project was in the middle of planning for step seven, the coronavirus disease 2019 (COVID‐19) pandemic hit, and restrictions prevented the project from being completed. As a result, this study only highlights steps one through six. While in the midst of this pandemic, the city of Louisville became the center of uprisings surrounding the murder of Breonna Taylor by officers in the Louisville Metro Police Department. Several of the youth Photovoice participants/YIT members became involved in organizing around justice for Breonna Taylor.

### Participants

Ten research participants from the YIT's 18 members agreed to participate in this study. At the outset of the Photovoice project, the youth self‐selected into one of two teams: (a) the photo‐takers (*n* = 7), or (b) the data analysts (*n* = 3). The original intention was for the photo‐takers' responsibility to end before data analysis began, but they all ended up wanting to contribute to data analysis as well. In the end, there were three youth who contributed to data analysis without taking photos, and seven who contributed to both phases of the project. To maintain confidentiality of the participants, we did not collect demographic data on those who participated. We do know, however, that most of the study participants were youth of color and lived in the West End.

### Procedure

The relationship between the university‐based research team and the YIT began through an interpersonal connection between one of the faculty leading the project and the YIT director. When the university research team received funding to conduct this study, the researcher asked the YIT director whether there might be any interest in collaborating. After consulting with the full youth leadership team, it was clear that the goals of the team aligned with the goals of the researchers, and we initiated a collaboration. The goals were to amplify youth voices and advocate for social change. The Photovoice work was separate from the regular work of the YIT, with all project meetings occurring outside their regular meeting times. The youth who participated in the Photovoice project ranged in their experience with the YIT; some had been active members of the team for multiple years, and others were more sporadic attendees who were in their first few months on the team. The university research team included four faculty members, one doctoral student, and approximately 10 undergraduate students. The number of undergraduate students fluctuated across the span of the project, depending on the needs at the time. Undergraduate students contributed in a variety of ways: some taught YIT participants how to use cameras; some transcribed audio recordings; some assisted in the Photovoice training; some contributed to data analysis; and some were involved in multiple areas of the project. The study was approved by the university's institutional review board and all participants provided consent/assent, including guardian consent among participants under age 18. All authors complied with American Psychological Association ethical principles in their treatment of and engagement with participants and their data in this research project.

To prepare the team for this collaboration, one of the faculty members led a series of workshops on Photovoice and qualitative data collection. To build relationships with the youth, the research team would often attend the last 30–60 min of the YIT's regular Saturday morning meetings, listening quietly and learning about the team's goals and priorities. At the end of the meetings, the research team provided lunch and began conversations about the Photovoice project among the YIT members who wanted to participate. We also hosted one meeting on the university campus at the request of the YIT members. One of the faculty members gave a presentation on Photovoice to the youth leadership team and facilitated a discussion of what the youth would like to investigate using Photovoice. Ultimately, they came to a consensus around the question of “What's it like in my ‘hood?,” where they would photograph salient features of their neighborhoods (i.e., their “hood”) and how those features shaped their lives.

After the youth decided on this topic, they were provided digital single‐lens reflex cameras and received hands‐on training from photography students on how to operate them. They also received training on Photovoice best practices, including avoiding taking photographs that included people's faces or property without asking permission. After being trained in using the cameras and the Photovoice process, seven youth spent the next month in their neighborhoods taking photographs, selecting five for which they would write captions and discuss with the group. The SHOWeD method was used to prompt reflection and analysis of the selected photographs; questions included: *What do you **See** here?, What is really **Happening** here?, How does this relate to **Our** lives? **Why** does this concern or situation exist?, How can we become **Empowered** through our new understanding? and What can we **Do**?* (Hergenrather, 2009). The photographs, captions, and transcripts from these sessions constitute the data analyzed for this study.

### Data analysis

As prior research indicates, YPAR exists along a continuum of youth involvement across various aspects of a research project (Anyon et al., 2018). In the data analysis phase of this project, we invited youth participation in all stages of the analysis and received interest in some of those stages. To begin the analysis process, the data analysis team, comprising faculty and graduate research assistants from the university, followed a team‐based thematic data analysis process (Braun & Clarke, 2012; Nowell et al., 2017) that focused on synthesizing the themes identified by the youth leadership team, while also allowing for other themes to emerge organically through the process. We began by creating a codebook, iteratively identifying and making tweaks to the themes and their definitions. Next, we applied the codebook to the data, ensuring each piece of data was coded by two individuals. Coding was guided by the constant comparative method (Glaser, 1965), in which coded data were constantly compared with both (a) the definition in the codebook and (b) previously coded data to ensure consistency in how the codes are applied. The purpose of this stage was to identify parts of the data that were relevant to a given theme, so we focused on being inclusive rather than endeavoring for inter‐coder reliability. After this, we focused on understanding the content of each theme. As with much qualitative research, we explored not just the most common themes, but also contradictions in the data, variability within themes, and connections across themes. The university‐based research team then engaged in member checking with the YIT who affirmed nearly all of what was presented and provided additional thoughts and context about several themes. The findings presented below, then, represent the meaning that the YIT made of their own photographs, as synthesized by the university‐based research team, and further informed by the YIT.

These themes and results of the member checking were used to inform the results and discussion of this manuscript. At the conclusion of the project, the university‐based research team explained to the youth participants our intention to write an academic journal article summarizing the project and invited any interested participants to contribute. None were interested, perhaps because they did not see any direct benefit to themselves, either because of the limits of our explanation or because there really may have been no benefit.

## FINDINGS AND DISCUSSION

The following sections present the themes identified around YIT members' articulation of community problems and solutions. In addition to presenting direct quotes, we also include youth‐generated photographs and captions that represent each theme. We integrate literature to draw meaning from our findings and theorize where these young people are in the stages of SJYD and critical consciousness development.

### What are the community problems from youths' perspective?

We identified three main themes regarding youth perceptions of the problems in their community. With a common thread of historical and structural racism, youth perceived the root cause of these problems as having a connection to public policy and organizational level changes with a specific focus on the city of Louisville. The themes include: (1) community divisions, disconnection, and structural isolation, (2) neighborhood neglect and abandonment by people in power, and (3) physical community conditions and consequences.

#### Theme 1: Community divisions, disconnection, and structural isolation

This theme centered the lack of power of individuals and groups to control how their communities are changing. Participants discussed inequities throughout their community, specifically those imposed by government entities and officials, that can restrict access and freedom, and fuel disconnection within and between neighborhoods. One participant explained how they observe the impacts of physical separation caused by gentrification,

Well, [as for] the “who,” I think government causes the divide. Only because it's like they‐[pause] I'm trying to tie it to gentrification. [resumes idea] It's like they take us and put us in these places where we're cut off from, like, the, probably the rest of the city. So like, with the West End, you don't have really much areas of [pauses] I guess social awareness? Um, like downtown you have all these different types of people, and all these collectives, and it's friendly. On the West End, hmm. It's complicated. ‐ Hero

In this quote, Hero problematizes how the city's policies have left the West End isolated from much of the rest of the city, including nearby neighborhoods that experienced revitalization. Although Hero contrasts West End residents' lack of “social awareness” with the more collectively oriented and friendly downtown, they identify the source of this disconnection as rooted in local government policies.

Another participant explained the impacts policies, local businesses and other entities have on their ability to build connections with their friends and other individuals in their community. For example, a local mall in Louisville has a policy that states: “We require that those 17 and under be accompanied at all times by a parent or supervising adult aged 21 or older after 4 pm on Fridays and Saturdays.” Nile explained the impacts of policies like this,

So now it's like we're limiting the interaction of us going out with your friends and it's lessening the connections that we're lacking. Like, the places that you used to go to hang out with your friends, we can't do it without an adult, or we have restrictions because somebody did something stupid, and now we don't want to go out because “Oh, I have to bring my parent with me” or I have a certain time that I have to leave because of something that my peer, that I don't even know, did. So, then it's like, those connections and the places that we had those connections are going away and so what are we supposed to do about it?

Captured within this theme were goals to shift the power from government officials to themselves to advocate for desired community changes. Amal explained the importance of staying connected as neighborhoods grow and change, “People are moving out of the neighborhood, new people are moving in, which is great, but I feel like the more my neighborhood grows, the less connected we become.” Another participant explained the importance of making sure people in their community had power to counteract the sense of hopelessness a lack of perceived power creates,

Like, there's a lot of things going on that our community believes they have no power of controlling, and we have all the power…We got the power, we just gotta move. Because life seem so hopeless. ‐ Baobab

**Figure 1:**
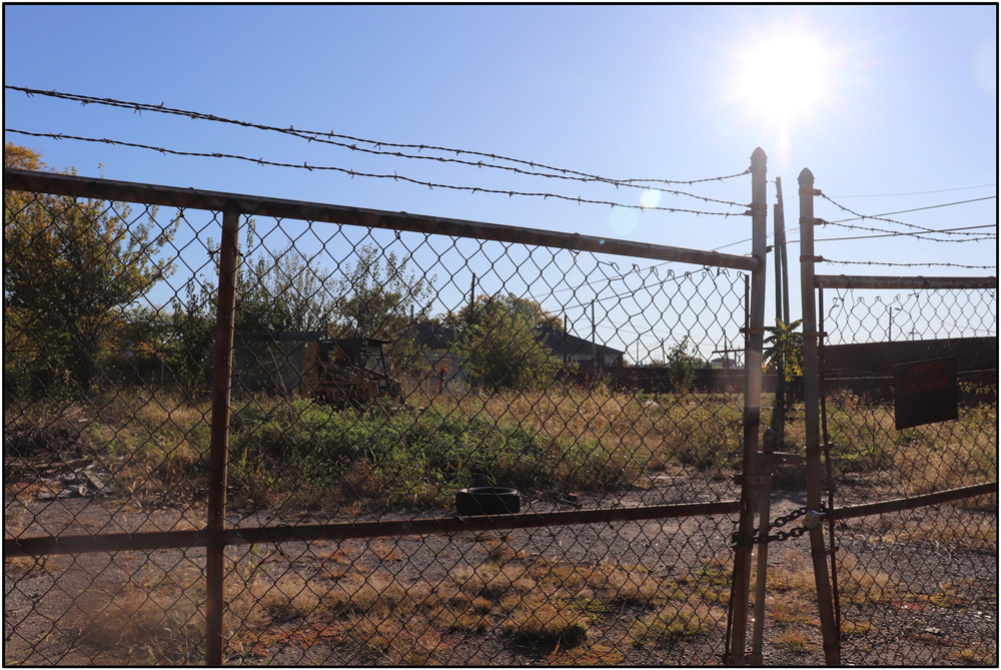
*“*I've realized that there is so much opportunity for change, yet people close off a way of change and get so tired. But as the visionary, I see that there is not enough time and that they are tired. Why are people so tired? People have things in their life going on and most of the time they don't have help of their own. As a community, we are not coming together as one to help and be in harmony with one another because there is a culture of not wanting others to know about our own individual hardships. As a nation, we are taught to have pride of self and not a heart to share with others. There is a fear and hatred that is a pollutant that can subdue a sound mind and turn a heart to stone, yet with this world you need to be careful of strangers. The division of people is leading communities to not move forward and is hindering us to build up a strong structure. So eventually instead of the project of community being under construction, there is a loss of construction that soon turns to debris.” ‐ Meadow.

Across this theme, participants described a separation or divide between neighborhoods and communities that is reinforced by structural changes and individual decisions beyond their control, like gentrification and policy shifts. As the composition of their neighborhoods change, participants observed an increased sense of disconnection and powerlessness. Figure 1 chronicles a young person's perspective on the ‘pollutants’ of hate and division, and how these pollutants are a result of people being closed off to change.

According to the literature, these observations exemplify a *critical reflection*, the first level of critical consciousness during which youth begin to notice the role of power in their worlds and develop a sensitivity to how that power shapes their everyday experiences (Watts et al., 2011). This awareness of power is also foundational to SJYD in which young people start to “contest, challenge, respond, and negotiate the use and misuse of power in their lives” (Ginwright & Cammarota, 2002). Participants' engagement with the Louisville YIT and exposure to the inner workings of local government may have provided background knowledge and language to describe some of the structural problems they are seeing. Their mention of control and power, as it relates to government leaders and their West End neighbors, suggests youth are grappling with who is responsible for addressing those problems.

#### Theme 2: Neighborhood abandonment and neglect by people in power

This theme illuminated youth perceptions of problems related to the abandonment of both people and buildings in their community. Captured within this theme are youth participants' discussions surrounding the lack of investment from government officials and businesses. One participant, Hero, discussed how people in power neglect and fail to draw attention to the conditions of their community,

… the West End is filled with a majority of houses that [are] abandoned and neglected. And, I mean, it's horrible. …imagine riding down the street and all you see is abandoned buildings. This isn't like, “what is wrong with this neighborhood” to where there's nothing wrong with it. There might be nothing wrong with it, but people with power sometimes neglect that there's places you know, kind of, are here and being taken advantage of. And they're not doing anything with it. ‐ Hero

**Figure 2:**
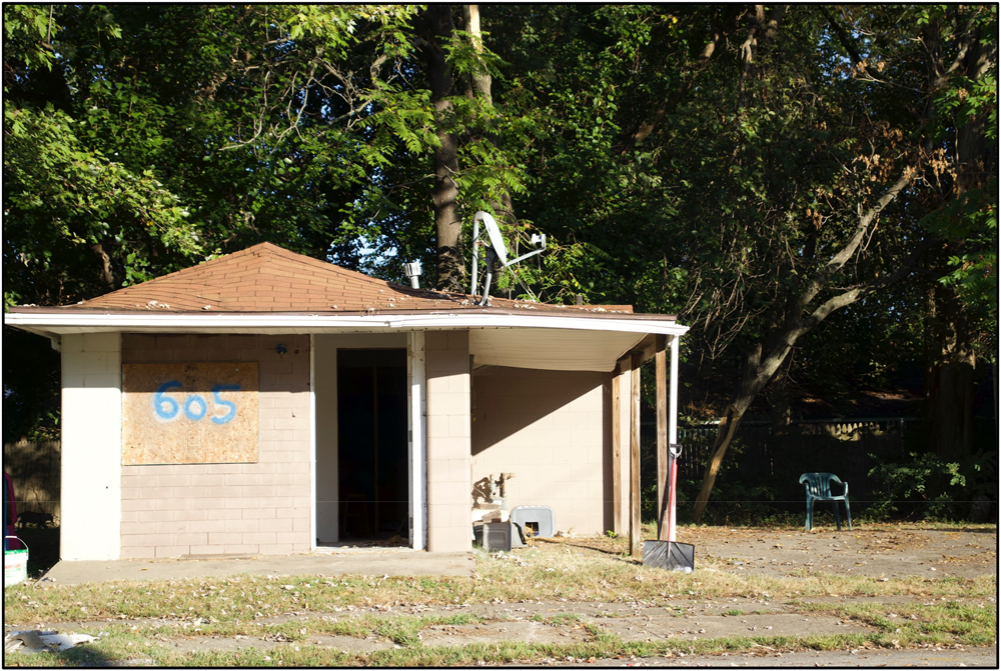
*“*I got abandonment issues to deal with, not from the people just the places that just made me feel it, the feeling of childhood and vivid memories, but now I look up, the Home is taken and no one can hear me, its been a long line of hurting from these terrible things, it's called gentrification, it made my people sing, but not out of joy, it's just out of trauma, the way they take our houses causes all of our drama, they feed us lies and then the disrespect, they make us move and we still are left with the debt, they make a killing out of us in fact, for when they don't ask, they just take it back, I'm tired of dealing with this damaged and unfaithful process, there's no way to leave without it taking your conscience.” ‐ Hero.

Youth participants pointed out that people were also abandoned in their community, not just buildings; Figure 2 shows an example of this connection. Meadow explained, “there's so many boarded up houses, so much trash, so much trash and, I don't know, disarray? …I feel like people are not only isolated but abandoned.” Youth participant Hero also attributed this isolation and abandonment to the effects historical policies like redlining have on certain communities such as Louisville's West End, “I feel like, kind of, companies and government neglecting the West End, ties back into redlining, gentrification, and everything.”

Miracle, another youth participant, observed a lack of urgency to address the consequences of historical policies that have segregated the West End and left it saturated with fast food restaurants without accessibility to high‐quality food options and grocery stores:

Like historically, these poor communities; they're effects of redlining. And, you know, they would only give loans and sell houses to Black and Brown people in these specific areas of town, like sort of modern segregation, and we're still feeling the effects of that to this day. And I don't know if it's still because of racial reasons, or because now like poverty and the economic inequality of these regions that are the problem now. But the city just doesn't invest as much in these areas, because there's less of a, you know, [begins a new thought] when you go out to like, richer areas, there's a Starbucks. Instead of Family Dollars there's like, Whole Foods. But if you come down here, like the nearest restaurants are Long John Silver's and McDonald's. That's a whole other can of worms, but it just shows that even the quality of food ‐ health related concerns ‐ it's just not equal.

Across this theme, participants acknowledged the lack of financial and personal investment in their neighborhood, not only by those who live there but by those elected to serve them. Participants explicitly named redlining, gentrification, economic inequality, and segregation as structural problems that have dire consequences on their health and wellbeing and result in feelings of abandonment, isolation, and neglect.

As young people notice the role of power, the question of *why things are as they are* becomes pressing. The literature states at the *critical reflection* or *self‐awareness* stage of SJYD, youth explore how elements of their social identities (e.g., class and race) connect to the social problems they face; this sensitivity is more common among young people whose marginalized identities are salient and central to how they see themselves and their communities (Kiang et al., 2021). Ginwright and Cammarota (2002) explain that this awareness incorporates an analysis of how “power, privilege, and oppression threaten their identities and capacity for self‐determination” (p. 89). Although this project did not assess identity salience among participants, their testimonies touch on how poverty, gentrification, and segregation are all linked to racism—indicating a perception that the conditions they describe are not caused by individual faults or failures but are the consequences of unjust and historical structures. This shift in blame counters the deficit narratives about underserved communities and directs collective attention toward policymakers and other executive decision‐makers, like city mayors.

#### Theme 3: Physical community conditions and consequences

In this final theme about community problems, youth highlighted various forms in which structural inequality has real consequences, shaping child and youth development in visible and tangible ways. Youth discussed seeing narcotics, addiction, homelessness, tobacco products, alcohol, and bloody needles. Miracle explained,

So, the trash that's in this photo, I don't know if you can see it, there's a beer can and then behind it is a pack of cigarettes. So, in my neighborhood, narcotics, [and] addiction, [are a] huge issue. That also ties with education and homelessness. Just think, if you're a little sixth grader getting off the bus, going home from school, you're walking, you're walking, your foot hits something. “Oh, it's a beer can!” You've never seen one of these before.

Another participant, Mountain, discussed the potential impacts of this exposure on her siblings,

I do have siblings. And when I think of things like [begins a new thought] I know that my brother went to a daycare on the south end of town. I remember 1 day we went to pick them up, and we looked on the grass and there were needles everywhere. Bloody needles on the ground. And that really just made me understand that people don't really care. People are getting exposed to things like that at the age of three, and even though they might not understand exactly what it means it's still there.

**Figure 3:**
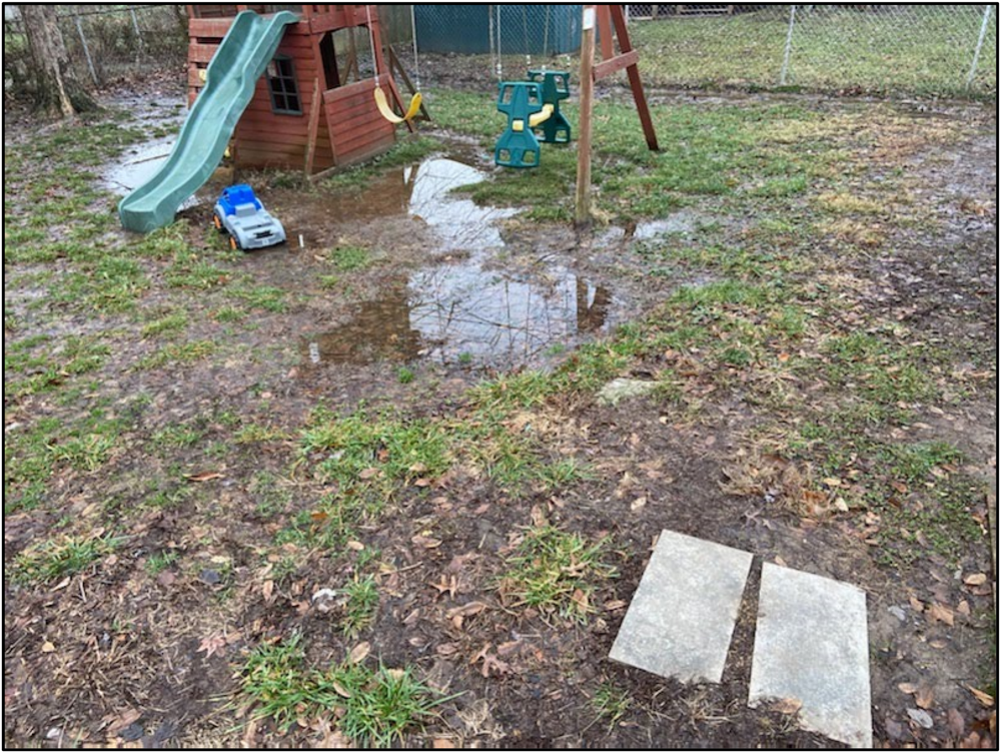
*“*To me this photo represents youth being interrupted. Bad weather often represents struggles and hardships in life. The puddle being near the playground makes me think of young people having to grow up too fast due to the struggles they're having to face at such a young age. The puddles seem to be in the way of potential happiness.” ‐ Mountain.

These two participants connected the physical disorder of their community—which elsewhere they linked to historical disinvestment—to harmful developmental consequences for young people, especially children. The narrative associated with Figure 3 describes the costs of having to grow up too fast, because of this exposure.

Youth participants also described how living in these environments puts them and others in danger. Youth acknowledged that with minimal resources to deal/adjust to life circumstances, they witness their communities experience mental health issues and a lack of coping skills. One youth participant, Baobab, shared a story about a friend (age 9) who witnessed his brother commit suicide. Baobab explained,

For instance, [remember] that friend of mine who watched his brother commit suicide in front of him? He always seen in a negative perspective. Which I understand. Your brother committed suicide in front of you at the age of nine. That's hard. But I wish I could get him the see it in a different perspective, as “Your brother was dealing with a lot. He committed suicide for a reason in hopes to be in a better place.” So all about him seeing the perspective of‐[rephrasing] You know how the pastor's be like, “We're not here today to mourn a death, but to celebrate a life,” you know? I started actually valuing that quote, because I want to celebrate their life, and hope that they're in a better place because this life? Sometimes to people it ain't nothing. They don't care about it, which I get. Horrible conditions. But I began to see in a different perspective. Not as death, but a second chance for a better place. And for our community, I would also hope for them to see it because death is common in our neighborhood. It is negative, but if we keep holding it in for the person that died as negative, suicide rates gonna continue. Way higher. And I hope this picture specifically has something of change in it.

Across this theme, participants identified the personal costs of living in a place that has been abandoned and neglected. Participants discussed the early exposure to drug use, inequality, and death, and the longer term consequences on their spirit. These young people acknowledged reducing these harms is difficult without structural changes *and* shifts in perspective. Participants also distinguished their points of view on the issues from those of their neighbors, signaling an awareness of the intricacies within a shared experience.

A sensitivity to the experiences of others, which we see here, is a significant developmental shift from egocentrism, as is the consideration of future generations (i.e., siblings, younger children). The SJYD model describes this shift as an advancement from a “self” awareness (i.e., how does X affect me) to a “social” awareness (i.e., how does X affect us; Ginwright & Cammarota, 2002, p. 94). The literature affirms that youth who experience marginality are uniquely capable of conceptualizing complex social problems and are driven to engage in social actions (Bañales et al., 2021; Tyler et al., 2020). Indeed, these competencies prime young people for the development of a critical consciousness (Watts et al., 2011). As youth transition from the *critical reflection* to *critical motivation* stage of critical consciousness development, they consider the responsibility and urgency to address those social conditions for the good of others.

### What are the solutions to community problems from youths' perspective?

In addition to themes around community problems, we also identified themes around solutions that would help address these problems. We have organized three themes: (1) build collective power through education and stronger networks, (2) multilevel change in attitudes and awareness, and (3) increase resources to support mental health and create spaces for youth.

#### Theme 4: Build collective power through education and stronger networks

This first theme focused on strategies for community building, education, and voting. Highlighting the importance of making sure communities know their voices are powerful rather than focusing on past historical events that caused the issues, Miracle explained,

I think the main issue right now, we shouldn't really be focused on the historical cause. We need to focus on what we can do right now. And I think what we need to do is lobby for change and just let the city know that this is a problem that we care about it. Like, no one in this part of town really recognizes that their voice is just as important as anyone else in the city. I feel like if I could change that, if I could, like, rally people, we could let officials know, “Hey, we're still here.” We have a voice and this is important to us.

Another collective power‐building strategy described by one of the youth participants focused on cultivating stronger networks. Amal expressed the potential power of connecting with their neighbors and showing care: “I don't know, I'm just one person! [Room laughs] But maybe we could start with doing those community block parties again, or going and talking to a new neighbor every day and seeing how they're doing and seeing what their lives are like.”

Ensuring their community had access to education on the importance of collective power building strategies, like voting were also discussed by youth participants. Miracle stated,

One of my projects and a mission of mine, I'd say, is to increase voter turnout and let people know. It's like a bystander effect if everyone thinks that their vote doesn't matter, like 90% of the neighborhoods like “Oh, just fine. Someone else. Oh, I don't need to go out.” Then everyone's silenced. So it's an important to learn. [It's a] behavior to break and I think education is how we can start.

In this theme, participants note critiquing and understanding the systems‐level causes are important, but can only do so much to improve the conditions of their neighborhood. They signal that even though awareness helps curb self‐blame and hopelessness, it is the responsibility of the community itself to recognize their voice and make change happen through collective and civic actions. Some solutions participants name here, such as block parties and talking to a neighbor, can build social ties and solidarity in the community, though are inherently located at the community/individual‐change level.

**Figure 4:**
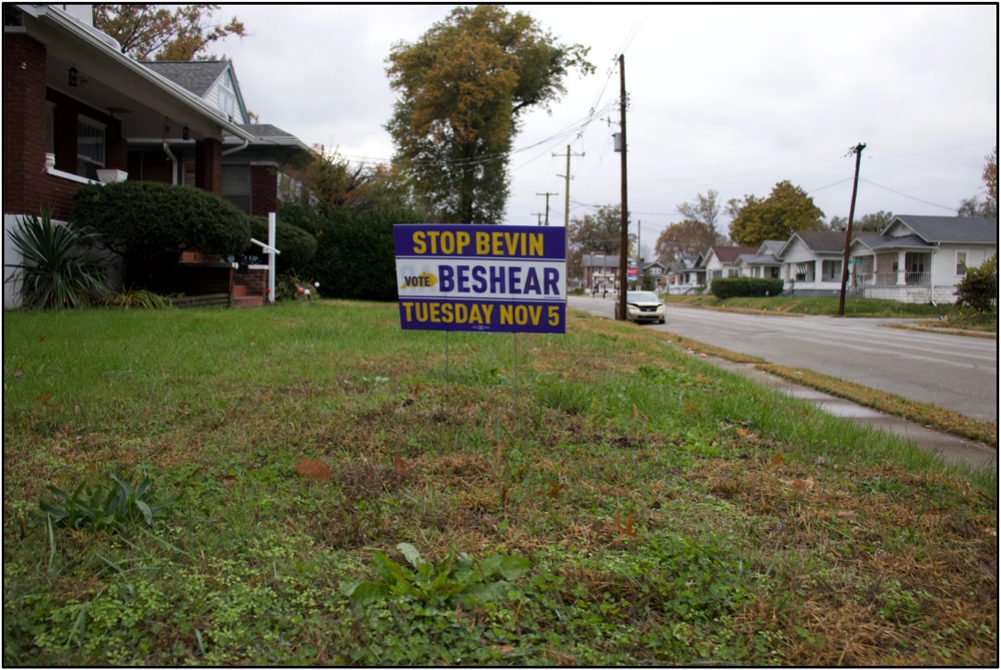
*“*Go to the polls; get out and vote! I see a political ad in a yard for Governor Beshear for the gubernatorial election. My neighborhood is predominately liberal/Democratic area, and they try to aid Beshear and get him more votes to get him into office. Government affects us all, whether it be local, state, or federal. It's important that everyone feels like they have a voice so that they're properly and accurately represented. Because we try to increase voter turnout. Anyone who walks by could be effected/inspired to go vote. Educate the people on the effect that they have on policy and their representatives.” ‐ Miracle.

Other solutions, like voting and speaking with city officials, are described by participants as having potential to address community conditions on a systems level. Figure 4 affirms that young people, even those who are not yet allowed to vote, are able to see the power of voting for improving community conditions and wish other West End residents would see how voting can represent the neighborhood's “voice.”

Participants' testimonies under this theme indicate they are at the *critical motivation stage* of critical consciousness, possessing a belief in one's individual capacity and responsibility to play an active role in social change (Watts et al., 2011). Their testimonies also suggest that in their neighborhood, a belief in the power of *collective* action needs to be rallied. A *community's* belief in their capacity to affect change has been described as collective efficacy (Bandura, 2000; Sampson, 2008). The literature affirms communities with strong social ties to each other—whose neighbors care for and trust one another—have greater collective efficacy and are more willing to participate in collective actions (Collins et al., 2014; Sampson, 2008). Although our youth participants are only a small sample of Louisville residents, the hope they communicate is palpable.

#### Theme 5: Multilevel change in attitudes and awareness

The next theme captured the importance of building awareness within and outside their community to shift negative perceptions and attitudes about the West End. People with power, such as the mayor, and those with White racial identities were identified as individuals who need to be more aware of lived experiences in the West End to create impactful change. Hero stated,

I feel like the people that live there need to know about this. Also, people that, like, are in the higher powers like the Mayor's office, or something similar. To actually make a change, instead of just knowing about it and just not doing nothing for it. I think, in general…people with power, and I want to say, just, I guess, more White people being aware of what we have to go through. Um, I just think if there's more awareness there can be proper change.

Youth participants also noted the importance of change in awareness and attitudes for those living in their communities too. Hero explained the critical role of holding a positive narrative about their community,

And just give it a better narrative. Because the West End is seen as a bad place where all you see is gun violence, and drug use, and, just, gloominess. That's the narrative that is posted. And especially within the Black people‐ [rephrasing] the African‐American community. So it's just changing the narrative and making it a better place for the youth to grow up in, and claim as their own. And in a proud way. To where they can end up being successful and keep on trying to make the West End a better place for them and their kid and stuff like that.

**Figure 5:**
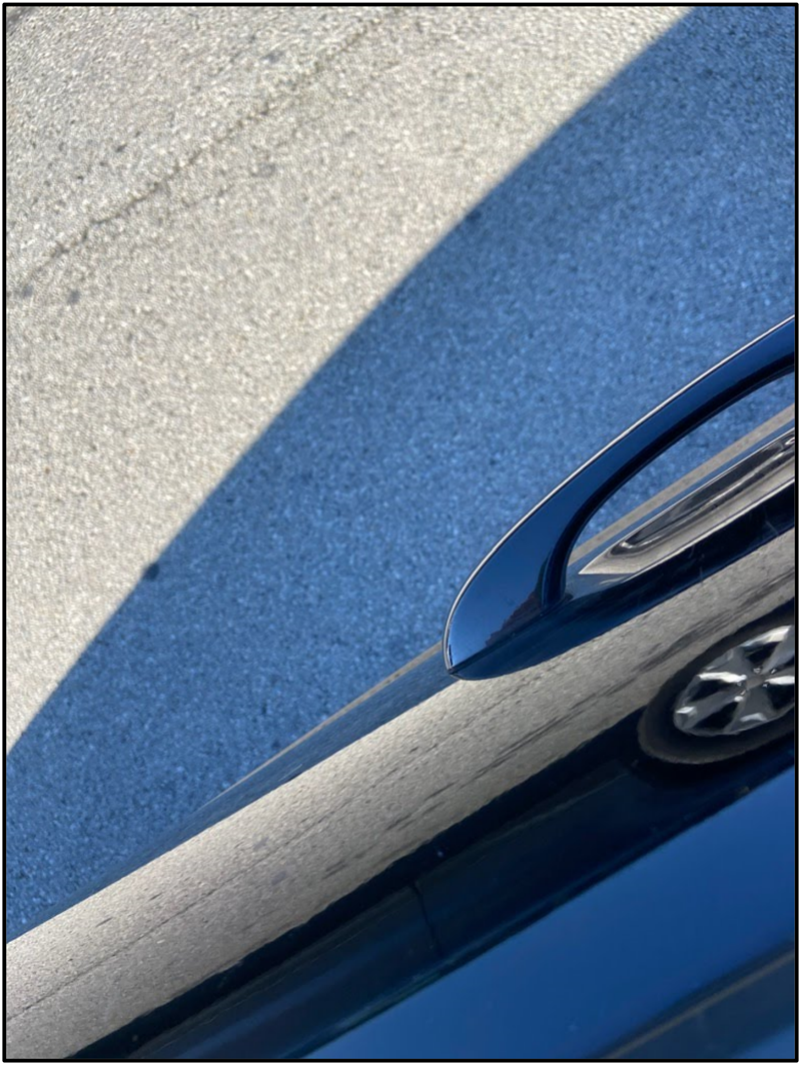
*“*This photo makes me think of the comparison between life and roads. Roads have bumps, curves, and sometimes holes just like life does. Those bumps take time to get over, those curves take time to get around, and those holes take time to get out of and fill. Roads take you on wonderful journeys. Life is a journey in itself, but whether or not it's wonderful depends on the drive and ambition of the person steering. The reflection represents perspective. Though you may see things one way, the world could look at them totally differently.” ‐ Mountain.

In the testimonies under Themes 4 and 5, participants discuss dynamics unique to White and Black communities, as they relate to specific narratives about their neighborhood. Youth clearly are familiar with what those narratives say and the harm they perpetrate in the West End. Participants seem to believe that when White Louisville community members try to learn about experiences of West End residents *and* their Black/African‐American neighbors critique and reject those narratives, it is possible to transform the West End to be a better place to live. Figure 5 notes the importance of this multi‐level shift. Participants' call‐to‐action directed towards different racial groups of Louisville residents shows they hope for a shared future with improved quality of life and connection for all.

Again, participants exemplify attitudes consistent with *critical motivation*, which is an awareness of their personal responsibility to reject harmful narratives and play a role in changing them (Watts et al., 2011). They communicate a hope for collective action and greater social cohesion between their neighbors and other Louisville residents. In the *social awareness* stage of SJYD, youth consider the alliances necessary to enact change (Ginwright & Cammarota, 2002, p. 88); our findings from this theme indicate alliances are needed across the city and that our participants believe change is not the sole responsibility of those most marginalized in Louisville.

#### Theme 6: Increase resources to support mental health and create spaces for youth

The final theme highlighted solutions that increase the resources available to community members, including investment in recreational spaces and community centers, community cleanups, and youth voice, organizing, and leadership. Having safe places to clear their minds were viewed as important solutions to participants. Baobab explained,

Like, for instance, the Boys and Girls Club. It's not as close to these neighborhoods as we would like, where kids can just go, you come off school, you had a bad day, you might have got into a fight. Here. Let me go over here and clear my mind and free it up and try not to worry and bring this back to my household and deal with it. More resources. Not to sound greedy, but actual resources to escape. Even our parks is dangerous, too, but even a place that kids can go hang out, and just be themselves, and feel free without having to be depressed or closed himself off so they could keep moving on to life.

Participants called for spaces for kids to be kids without trash and violence, where they can decompress and cope with stressors in their lives. In the absence of youth programs and clubs and especially in under‐resourced communities, young people often gather in public places like parks and playgrounds (Pryor & Outley, 2014). But even these settings become inaccessible when they are not maintained or are overtaken by adults. Our youth participants made a connection between these safe places and overall well‐being; a relationship affirmed by a variety of studies (Fleckney & Bentley, 2021).

Another solution identified by the youth focused on improving the physical conditions of their community to promote a sense of pride. Miracle stated,

…and I think we need to take pride in our community, take pride in our children and our students in a way that we don't really do nowadays. Like everyone, no one really takes pride in my community. That's how I feel when I look around and I see trash like beer, and McDonald's bags, and sewage everywhere. It's disgusting. And I don't think that it's fair that we don't take pride in ourselves, don't take pride in our community to really get out and just clean it up. Like, we could maybe go downtown to the mayor's office, or we could host a couple of community‐led clean‐ups, or something like that.

Participants identified that neighborhood neglect is also perpetuated by their own neighbors, not just by West End business owners and local officials. They noted pride in one's community takes commitment to foster, and once people feel proud of where they live, they are more likely to invest their time and money into that place.

Youth discussed the importance of investing in programs and initiatives that promote youth voice, organizing, and leadership. Miracle explained,

I think the reason I'm even doing this project is because I'm part of the Youth Implementation Team, I think organizations like that are very, very influential. And they're, like, a big, big motivation for people to get out there, and for kids to actually have a platform.

Youth also advocated for increasing mental health supports in their schools and communities. Mountain expressed,

I think that mental health is looked over a lot, and even though some schools are putting psychologists in the buildings now I just don't think that's enough. Sometimes they'll ask us how we feel, and we'll say we're okay or we're not, but it's not really looked into deep enough. Mental health is the main issue in like, everything. The way we think affects everything we do, and that's why the world looks the way does.

**Figure 6:**
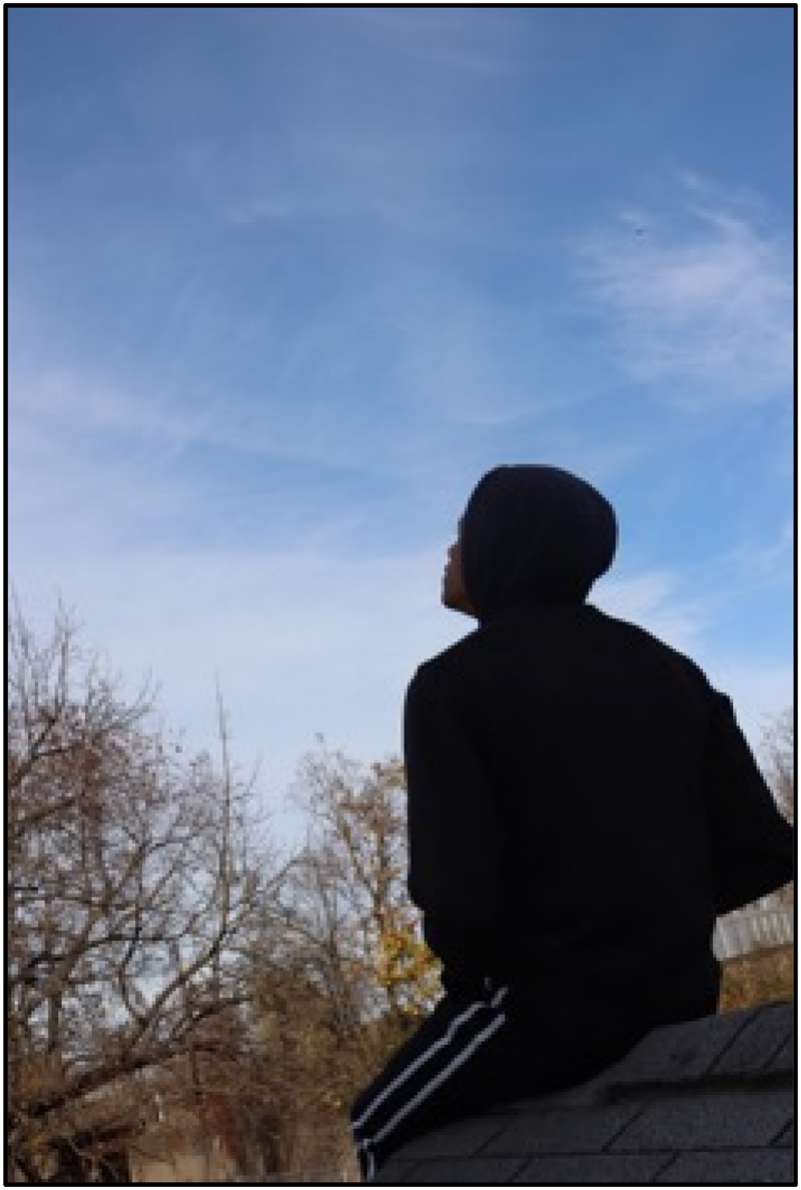
*“*This is an image of escaping to find peace of mind. A lot of people try to find an escape from reality but reality is the very thing we live in. We can't escape it until death and even that's unsure. We can't truly escape it but find a peace of mind in coping in reality. Some people escape through music, art, drugs, even harming others. Everyone needs that escape at times but what's really helping you escape or making it worse. We portray a self image of our self not knowing how some‐one else perceives you. Sometimes you need to find a space where you can let your conscience breathe even if it's just sitting on your roof like the person is in this photo. Mental problems are one of the most dangerous things people get killed by besides a bullet and we tend to feel disconnected searching for an outlet. Try to find your balance, outlet, or space to let your conscience breathe because this world is obviously not getting better especially with who's leading this world but that's a bigger picture.” ‐ Baobab.

Demands for mental health resources for young people and evidence of systemic barriers are well‐documented (Kentucky Youth Advocates, 2021; Radez et al., 2021). Community‐based organizations and programs that offer safe environments for youth to connect with peers and build solidarity over shared experiences can also meet their mental health needs and reinforce coping (Case & Hunter, 2012). The narrative for Figure 6 speaks to the value of creating space for young people to escape and find peace of mind. The use of participatory methods—like Photovoice—may be particularly useful to explore what kinds of settings are most helpful (Wallerstein & Duran, 2006; Wang & Burris, 1997).

### Summary

As their testimonies indicate across our six themes, participants are advanced in the *critical awareness* and *critical motivation* stages of critical consciousness (Watts et al., 2011). They named structural causes—disinvestment, redlining—and those they view as to blame. Participants also called out the harms and conditions perpetuated within their communities and were able to describe both individual (such as participation in the YIT) and community‐level actions (such as neighborhood clean‐ups)—hinting at *critical action* behaviors.

Participants do not describe structural solutions outside their realm of control or that of their neighbors (e.g., voting). This discord suggests that although their education about and exposure to local government through the YIT may have provided the language to describe structural harms, YIT members have not been directly involved in or witness to how structural change is *implemented* (e.g., instituting new policy). Alternatively, the discrepancy between who is responsible for problems versus solutions might indicate young people are sensitive to which changes are *realistic* and understand more judiciously the magnitude of systemic limitations and the resistance to change of those who govern their neighborhood. Our findings indicate participants feel frustrated with and a distrust of those who have the structural power (e.g., policymakers) to address the structural causes of community problems.

Youth in this study may perceive collective actions as more feasible and impactful in the short term, particularly when city leaders fail, refuse, or are too slow to use their political power to improve community conditions. It is also possible that the structure of the YIT program blurred the lines between individual or collective action and more structural changes. The YIT offered a direct line between Louisville youth and the mayor, with the intention of allowing young people a pathway to shape city‐level policy. Voicing their opinion or idea to a policymaker through the YIT might feel like an individual action; consequently, youth may not be cognizant of or privy to how this informs structural change over time (e.g., mayor instituting a resolution informed by those ideas). Youth in an initiative like the YIT may leave the program before they see the structural results of their individual actions, which may also influence their perspectives on structural solutions.

Based on our observations of the YIT and the findings from this Photovoice project, it is our opinion that the YIT's design mirrors the values of SJYD more than it does PYD. Incorporating education *and* collective action, YIT members clearly see themselves as social actors whose passions for social justice motivate their views and ideas. The YIT offers a setting for youth to ask pressing questions, connect with peers over shared experiences, and activate over issues that affect them, reflecting the values of participatory models (Kohfeldt & Langhout, 2012; Ozer, 2017). By extension, it reduces institutional and bureaucratic barriers so young people have real access to individuals whose decisions affect their daily lives— something few youth ever get to experience. We view the YIT as a contrast with PYD programming, which has traditionally focused much more on developing individual skills for thriving (in the context of often oppressive structures) without critically engaging youth in conversations about why those structures exist and how they might change.

While this study did not assess the individual‐level impacts on YIT members, evaluative data could assess the extent to which this model is worth replicating in other US cities. In addition, there are several useful tools, beyond Photovoice, governmental programs could utilize to support dialogue around social problems and solutions with young people of different ages (e.g., the Five Whys method; Kohfeldt & Langhout, 2012). Further research is needed with youth who are not engaged in initiatives like the YIT to see how they would answer these same questions, to assess if and how membership in the YIT is uniquely supporting critical consciousness development and SJYD. On the community scale, conversations between adults and youth residents may help researchers and policymakers alike understand how “change” is perceived by different groups living in the same place and what change they believe is needed.

## IMPLICATIONS FOR POLICY AND PRACTICE

To meet the ultimate goal of the Photovoice process, which is to reach policymakers and facilitate systems‐level social change, it is imperative that young people have a genuine platform to share their perspectives and ideas. This aligns with policy and practice recommendations from Bloomer et al. (2023) that include the following: (1) prioritize funding to enhance social justice youth programs and build spaces where youth can have a voice, (2) develop and implement youth programs to address mental health and well‐being, and (3) shift from individual‐level to structural‐level youth interventions.

Furthermore, it is often executive decision‐makers (e.g., mayors, city councils) who prioritize and dedicate funding to programs like the YIT, which create pathways for youth to learn about and be part of sociopolitical processes. Ultimately, young people need adult allies who are not only willing to open doors to spaces where decisions are being made but to pass the microphone to youth who wish to voice their experiences and needs. Programs that do so are not only more likely to support young people's development of critical consciousness but expand possibilities for them to inform, guide, and witness structural change (Zeldin et al., 2013).

Policymakers must not only show up in the communities they serve but also listen to those most directly affected by their policy decisions, particularly those who are traditionally excluded from the policymaking process, such as young people. Forging a line of communication and reciprocity between community members and policymakers can foster trust and encourage civic engagement (Bloomer et al., 2023) and might be the key to envisioning and implementing structural changes that improve quality of life and create more equitable communities. If not to do that, what is the real intention of engaging youth in local government?

## CONCLUSION

Although the youth participants were adept at understanding the structural underpinnings of the community problems they identified (e.g., racism, gentrification, redlining), their solutions largely included actions situated at the individual or community level (e.g., voting, mental health supports, community cleanups). Viewing these results through the lenses of critical consciousness development and social justice youth development, our findings suggest the youth in this study may view systems‐level solutions (like policy changes) as unrealistic, or they may not know what those solutions could be or look like. Regardless, if young people who are members of a mayor‐supported youth coalition that incorporates both education and action struggle to name or visualize structural solutions for well‐documented community issues like violence and poverty, what chance do other youth or adult residents have who do not have that same experience and exposure?

## CONFLICT OF INTEREST STATEMENT

The authors declare no conflicts of interest.
